# Water Hardness Can Reduce the Accumulation and Oxidative Stress of Zinc in Goldfish, *Carassius auratus*

**DOI:** 10.3390/antiox11040715

**Published:** 2022-04-05

**Authors:** Cheol Young Choi, Zhongze Li, Jin Ah Song, Young-Su Park

**Affiliations:** 1 Division of Marine BioScience, Korea Maritime and Ocean University, Busan 49112, Korea; lzzz@g.kmou.ac.kr; 2 Marine Bio-Resources Research Unit, Korea Institute of Ocean Science and Technology, Busan 49111, Korea; jinah@kiost.ac.kr; 3 Department of Nursing, Catholic University of Pusan, Busan 46252, Korea; yspark@cup.ac.kr

**Keywords:** antioxidant, apoptosis, goldfish, toxicity stress, water hardness, zinc

## Abstract

We investigated the changes in toxicity stress in goldfish, *Carassius auratus*, under exposure to different concentrations of Zn and water hardness for 14 days. We analyzed the changes in water hardness and Zn accumulation after exposure. To investigate the stress levels, the expression of metallothionein, caspase-3 activity, NO activity, and total antioxidant capacity were detected. Terminal deoxynucleotidyl transferase dUTP nick end labeling (TUNEL) assays were also performed to measure apoptosis in the liver. The results showed that compared to the control group, a more significant difference in the accumulation of Zn in body stress markers (metallothionein, caspase-3 activity, NO activity, and total antioxidant capacity) were observed with increasing Zn concentration and exposure time. Notably, at the same Zn concentration and exposure time, lower stress levels were discovered in the samples under harder water conditions. Finally, the TUNEL assay showed that Zn accumulation caused apoptosis and high water hardness could reduce the apoptosis. In conclusion, we found that high water hardness can influence the absorption of Zn, and alleviating the hardness levels can reduce the toxicity stress caused by Zn.

## 1. Introduction

Industrialization has led to the occurrence of various pollutants, as industrial wastes containing high concentrations of heavy metals are discharged into the aquatic ecosystem, increasing their concentrations in the environment [1,2]. Pollution caused by heavy metals has been a concern for several decades. Most aquatic organisms are directly or indirectly affected by the chemical composition of water [3]. Moreover, most heavy metals, including essential and non-essential elements, are not biodegradable and accumulate in the fish body because of their long biological half-life [4]. Bioaccumulated heavy metals act as stressors to aquatic organisms and inhibit the synthesis of proteins and nucleic acids, causing various metabolic, biochemical, physiological, and histological changes [5,6]. 

Among various heavy metals, Zn is an essential trace element in the body for the synthesis of cellular enzymes [7]. However, when fish are exposed to high concentrations of Zn, toxic responses, such as the destruction of various protein structures, are induced in their bodies [8,9]. According to a previous study [10], the concentration of Zn increased to a high level around the world from the 1970s (0.23 ± 0.47 mg/L) to the 2010s (0.48 ± 1.38 mg/L). Excess heavy metals may release reactive oxygen species (ROS) and reactive nitrogen species (RNS), leading to oxidative stress [11]. In particular, representative ROS include H_2_O_2_, OH-, and O_2_- while RNS include NO and NO_2_ [12]. When the concentrations of ROS and RNS are high in the body, peroxidation of proteins, lipids, and nucleic acids occurs and sometimes apoptosis is induced [12]. 

When an organism is poisoned with heavy metals, including Zn, metallothionein (MT) is activated in the body as a detoxification mechanism. MT serves to offset the toxicity of heavy metals by combining with various heavy metals, such as copper, Zn, and Hg [13,14]. Additionally, MT plays a role in inducing an antioxidant response to remove excess ROS [15]. The antioxidant response mainly occurs in the liver, and the ROS that is not removed through the antioxidant response process is known to cause inflammation. Typical examples of genes causing inflammation include nitric oxide synthase (iNOS) and cyclooxygenase-2 (COX-2) [16,17]. When a fish is exposed to a toxic environment, the expression of iNOS, which is involved in NO production, is induced in the fish body. Nitric oxide (NO) is a representative oxidative substance. The excessive production of iNOS causes apoptosis by promoting oxidation of cells and excessive induction of NO [18]. Enzymes involved in apoptosis include caspase (cysteinyl aspartate-specific proteinase), of which, caspase-3 plays a role as an enzyme that catalyzes cell protein degradation and apoptosis [19]. 

The water quality of an aquatic environment, determined by parameters, including pH, water temperature, oxygen content, and hardness, is very important for the survival of aquatic organisms [20]. Among these indicators, hardness can be defined by the amount of polyvalent cations but most often by the concentration of Ca^2+^ and Mg^2+^, which are commonly present in the natural environment [21]. Therefore, hardness can generally be measured by the concentration of CaCO_3_ in water. Usually, water containing 0−90 mg/L CaCO_3_ is considered soft water, 90−350 mg/L as hard water, and >350 mg/L as very hard water. A previous study [22] reported that water hardness had an effect on the metabolic activity of the South American catfish, *Rhamdia quelen*. Another study [23] showed that the growth rate of the common snook, *Centropomus undecimalis*, was the highest in the test group containing water with 100 mg/L CaCO_3_ hardness. Furthermore, a study on the degree of acute toxicity through the survival rate of fish exposed to various heavy metals reported that the higher the water hardness, the higher the lethal concentration (LC50) for heavy metals [24]. These results suggest that the toxicity of heavy metals can be inhibited by increasing the water hardness. A study by Baldisserotto [20] reported that Ca^2+^ inhibits the absorption of heavy metals by reducing the penetration of heavy metals into the gills. Alsop [25] reported that the amount of Zn^2+^ dissolved in water decreased as the hardness increased when ZnSO_4_ was added to the hardness test plots of various concentrations. 

Generally, compared to seawater, fresh water has low ionic strength, which increases the saturation concentration of external pollutants, and thus, increases the risk of heavy metal (such as Zn) contamination [26]. 

Therefore, the possibility of reducing toxic stress in fish must be investigated by controlling the water hardness caused by Zn. 

In our study, we selected a species of goldfish, *Carassius auratus*, which is a freshwater fish mainly used for toxicity evaluation due to its low sensitivity to toxic substances, and exposed it to various concentrations of Zn and water hardness, to determine the stress factors and the degree of apoptosis. According to a previous study [27], the water hardness in Korea river is around 50~90 mg/L, which is soft water and the hardness can thus be increased. In addition, we analyzed the effects of these two parameters (Zn concentration and hardness) on the physiological response of goldfish to examine the extent to which hardness reduces the toxicity of Zn.

## 2. Materials and Methods

### 2.1. Experimental Fish

Goldfish, *C. auratus* (*n* = 384, body length 6.8 ± 0.8 cm, and mass 12.4 ± 2.1 g), were purchased from Choryang aquarium (Busan, Korea), acclimatized for 1 week, and divided into control and experimental groups. They were maintained in tanks filled with 300 L of fresh water in the laboratory (16 fish per tank). Four fishes from each replicate treatment group were randomly sampled at each time period. All experimental conditions had two replicates to ensure consistency. The freshwater temperature was maintained at 19.5 ± 1 ℃ with aeration and the feeding was stopped 24 h before the experiment. We used these vertebrates (goldfish) according to the animal study protocol approved by the Institutional Animal Care and Use Committee of Korea Maritime and Ocean University (Approved protocol no # KMOU IACUC 2022-02).

### 2.2. Zn and Water Hardness Treatment and Sampling

Goldfish in the experiment were exposed to Zn (ZnSO_4_, 83265, Sigma, St. Louis, MO, USA) concentrations of 0, 0.5, 1, and 3 mg/L and water hardness by adding CaCO_3_ (239216, Sigma, St. Louis, MO, USA). The concentration of Zn was set according to previous studies [8,28], which showed that a Zn concentration higher than 6.0 mg/L could lead to the death of the goldfish while 1.0 mg/L of Zn could cause oxidative stress. The control group for the water hardness was 90 mg/L CaCO_3_, according to the hardness value of running water in Busan, Korea detected using a water hardness pure water meter (PWH-303, Lutron, China). We considered 3 conditions of water hardness: soft water (S) (90 mg/L of CaCO_3_), hard water (H) (270 mg/L of CaCO_3_), and very hard water (V) (450 mg/L of CaCO_3_). For convenience, the experimental groups were named as follows according to the concentration of Zn (in brackets): in the soft water (S) group: Zn 0 + S (0 mg/L, control) Zn 0.5 + S (0.5 mg/L), Zn 0.5+ S (1.0 mg/L), and Zn 3.0 + S (3.0 mg/L); in the hard water (H) group: Zn 0 + H (0 mg/L), Zn 0.5 + H (0.5 mg/L), Zn 0.5+ H (1.0 mg/L), and Zn 3.0 + H (3.0 mg/L); and in the very hard water (V) group: Zn 0 + V (0 mg/L), Zn 0.5 + V (0.5 mg/L), Zn 0.5+ V (1.0 mg/L), and Zn 3.0 + V (3.0 mg/L). During this time, water hardness was detected and recorded daily. Before sampling, all the fish were anesthetized using clove oil (C8392, Sigma, St. Louis, MO, USA). Blood was collected rapidly from the caudal vein using a 1 mL syringe coated with heparin. Plasma samples were separated by centrifugation (4 °C at 12,000 × *g* for 12 min). Prior to analysis, the samples were stored at −80 °C and part of the liver samples was stored at 18–20 °C in 10% formalin. During the experiment, the mortality rate was 0% in all experiment groups.

### 2.3. Zn Accumulation in Fish Body

The fish were added to 60% NO and 2% of NO with 5 ppm of Au and decomposed at 100–150 °C for more than 12 h. Then, the mixture was filtered using Whatman No. 4 (WHA1004125, Sigma, St. Louis, MO, USA) filter paper and diluted using 2% nitric acid to a final volume of 50 mL. All samples were detected using an optical emission spectrometer (ICP-OES; Optima 2000DV, Perkin Elmer, Waltham, MA, USA) at an absorbance of 213.856 nm.

### 2.4. Expression of the Metallothionein Gene and iNOS mRNA

Total RNA was extracted from the liver using TRI Reagent^®^ (TR188, Molecular Research Center, Inc., Cincinnati, OH, USA), according to the manufacturer’s instructions. The purity of all the RNA samples was determined by the ratio of their absorbance at 260 and 280 nm (A260/A280), which was confirmed to be between 1.8 and 2.0. Total RNA (2 μg) was reverse-transcribed to complementary DNA (cDNA) using an oligo-(dT)15 anchor and M-MLV reverse transcriptase (RT0015, Takara, Japan) according to the manufacturer’s protocol. All the synthesized cDNA was diluted by 1:100 and stored at −20 °C. The relative expressions of MT, iNOS, and β-actin were measured by real-time quantitative polymerase chain reaction (qPCR). The qPCR primers were designed according to the known sequences at NCBI (Table 1). qPCR amplification was conducted by a Bio-Rad iCycler iQ multicolor real-time PCR detection system (Bio-Rad, USA), and the iQ SYBR green supermix (Bio-Rad, USA), according to the manufacturer’s instructions. As an internal control, the experiments used β-actin (accession no. LC383464) after evaluation by Bestkeeper from 3 reference genes, including β-actin, GAPDH (accession no. KT985226.1), and ribosomal protein S18 (RPS18, accession no. XR_003291850.1). The highest correlation among them was used as the internal control gene. All data were expressed as changes with respect to the corresponding calculated β-actin cycle threshold (ΔCt) levels. The calibrated ΔCt value (ΔΔCt) for each sample and internal control (β-actin) was calculated using the following equation:

ΔΔCt = 2^−(ΔCt sample−ΔCt internal control)^ (1)

All the results for MT and iNOS mRNA in the target genes were the relative values between the target gene and internal control (β-actin).

### 2.5. NO Activity and Total Antioxidant Capacity (TAC) in Plasma and Caspase-3 Activity in Liver

The NO activity was measured by an assay kit (BM-NIT-200 BIOMAX Inc, Seoul, Korea) according to the manufacturer’s protocol and the absorbance was measured at 450 nm. The assay kit can detect both NO_2_- and NO_3-_ to calculate the total activity of NO, which is one species of RNS. TAC was measured using an assay kit (BM-TAC-200 BIOMAX Inc, Seoul, Korea) according to the manufacturer’s protocol and the absorbance was measured at 540 nm. TAC was measured based on the Trolox equivalent antioxidant capacity and the assay is based on the reduction of copper (II) to copper (I) by antioxidants, which reflects the level of ROS. The caspase-3 activity in the liver was detected using an enzyme-linked immunosorbert assay kit (MBS012786, Mybiosource Inc., San Diego, CA, USA) according to the manufacturer’s protocol.

### 2.6. MT mRNA In Situ Hybridization

The MT sequence for the in situ hybridization probe was designed for the forward primer 5′-ATG GAT CCC TGC GAT TGC GC-3′ and reverse primer 5′-TCA TTG ACA GCA GCT GGA GC-3′ (Accession no. X9727.1), amplified using PCR, and ligated to a pGEM-T easy vector (A137A, Promega, Madison, WI, USA). Furthermore, the anti-sense was confirmed by sequencing, and plasmid DNA was amplified using PCR, with the antisense and T7 primer (5′-TAA TAC GAC TCA CTA TAG GG-3′). Digoxigenin (DIG)-labeled probes were created using a DIG RNA Labeling Mix (Merck, Darmstadt, Germany) and the PCR products using the anti-sense primer and T7 RNA polymerase were used as the antisense labeling probes.

The liver tissue of the groups (control, Zn 3.0 + S, Zn 3.0 + H, and Zn 3.0 + V) exposed for 7 days were stored in 4% paraformaldehyde (PFA) and in 30% sucrose to prevent frostbite before sectioning. Sections were hybridized with hybridization buffer (containing 5 mL of deionized formamide, 2.5 mL of 20× saline sodium citrate (SSC), 100 μL of 0.1% Tween-20, 92 μL of 1 M citric acid (pH 6.0), and DEPC-H_2_O up to a total volume of 20 mL), spiked with yeast total RNA (50 μL) and the RNA probe, and kept overnight at 65 °C. 

For hybridization signal detection, tissue sections were first incubated with a blocking solution (10% calf serum in 1× PBS containing 0.1% Tween 20 (PBST)) for 1 h at 20 °C, followed by overnight incubation at 4 °C with an alkaline phosphatase-conjugated anti-digoxigenin antibody (1:2000 in blocking solution; Roche, Basel, Switzerland). After a series of washing steps (6 times for 15 min each, in PBST at room temperature) and rinsing in alkaline Tris buffer, consisting of 1 M Tris at pH 9.5, 1 M MgCl_2_, 5 M NaCl, and 10% Tween-20 (3 times for 5 min each at room temperature), color imaging was performed using a labeling mix (1 mL of alkaline Tris buffer, 4.5 μL of nitroblue tetrazolium, and 3.5 μL of 5-bromo-4-chloro-3-indolyl phosphate disodium salt), which was sprayed over the sections.

Then, the sections were kept in a dark and humid chamber for at least 8 h to develop color. The slides were washed with PBST, fixed with 4% PFA for 1 h, mounted with Aquamount (Aqua Polymount, Warrington, PA, USA), and covered with a slip. A stereomicroscope (Eclipse Ci, Nikon, Tokyo, Japan) was used to capture the images. 

### 2.7. Terminal Transferase dUTP Nick End Labeling (TUNEL) Assay

A TUNEL assay was performed on the Zn 3.0 S, Zn 3.0 H, and Zn 3.0 V groups on day 14. Samples of liver were washed and fixed in 10% formalin using an ApopTag^®^ Peroxidase In Situ Apoptosis Detection Kit (S7100, Sigma, St. Louis, MO, USA) following the manufacturer’s specifications. Finally, all the slides were observed using an optical microscope (Eclipse Ci). Brown cells that could be observed indicated apoptosis.

### 2.8. Statistical Analysis

All data were analyzed using SPSS version 25.0 (IBM SPSS Inc., Armonk, NY, USA). For all parameters analyzed, a three-way ANOVA followed by Tukey’s post-hoc test was performed to compare the differences among the different Zn concentrations, water hardness levels, and exposure times of the samples, and the effects of the interaction among the three factors are shown in Table 2. The significance level was set higher than 95% (*p* < 0.05). The measured values are expressed as the means ± standard deviations (SDs).

## 3. Results

### 3.1. Changes in Water Hardness

No significant changes were observed in the hardness levels of the water with increasing exposure times in the experiment. After measuring the water hardness daily, we found that the soft water, hard water, and very hard water groups at various Zn concentrations retained the water hardness levels of approximately 90, 270, and 450 mg/L CaCO_3_, respectively.

### 3.2. Changes in Zn Accumulation in Fish

Zn accumulation was significantly affected by the exposure time, Zn concentration, water hardness, and all their interactions (Table 2). An increase in Zn accumulation occurred in the fish body after they were exposed to Zn (Table 3). The Zn 3.0 group showed a significantly higher accumulation than the other groups. After 14 days, the accumulation of Zn reached the highest level. Zn 3.0 + S reached 113.53 ppm, which was almost 300% of the value after 3 days of exposure. However, on day 14, the same Zn concentration groups, at higher water hardness levels, showed a significant decrease in Zn accumulation, with 90.07 (299% of Zn 0 + H) and 60.96 (249% of Zn 0+ V) ppm for Zn 3.0 + H and Zn 3.0 + V, respectively.

### 3.3. Changes in MT mRNA Expression in the Liver

**Figure 1:**
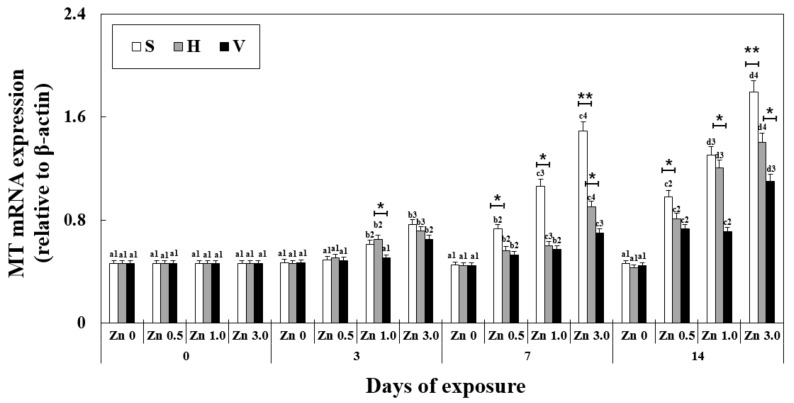
Changes in the mRNA expression of the metallothionein gene measured for 14 days. Different letters indicate significant differences among goldfish exposed to the same Zn concentration and water hardness. Different numbers indicate significant differences among goldfish exposed to different Zn concentrations at the same water hardness level and for the same duration. The symbol “*” and “**” represent statistical significance (*p* < 0.05 and *p* < 0.01, respectively) among goldfish exposed to different water hardness levels at the same Zn concentration and for the same duration. Values are mean ± SD (*n* = 4).

mRNA expression of the metallothionein gene was significantly affected by exposure time, Zn concentration, water hardness, and their interactions. The overall mRNA expression of the MT gene in the liver increased with increasing Zn concentration and exposure duration at the same water hardness level (Figure 1). Notably, at the same Zn concentration and exposure duration but increasing water hardness levels, the MT mRNA expression decreased (on day 14, the expression for Zn 3.0 + S and Zn 3.0 + V were 1.79 ± 0.19, 325% of Zn 0 + S and 1.10 ± 0.09, 199% of Zn 0 + V, respectively). Furthermore, no significant difference was observed between all the control groups with different water hardness levels and no Zn (0.46 ± 0.05). 

### 3.4. MT mRNA Expression in Liver Using In Situ Hybridization

**Figure 2:**
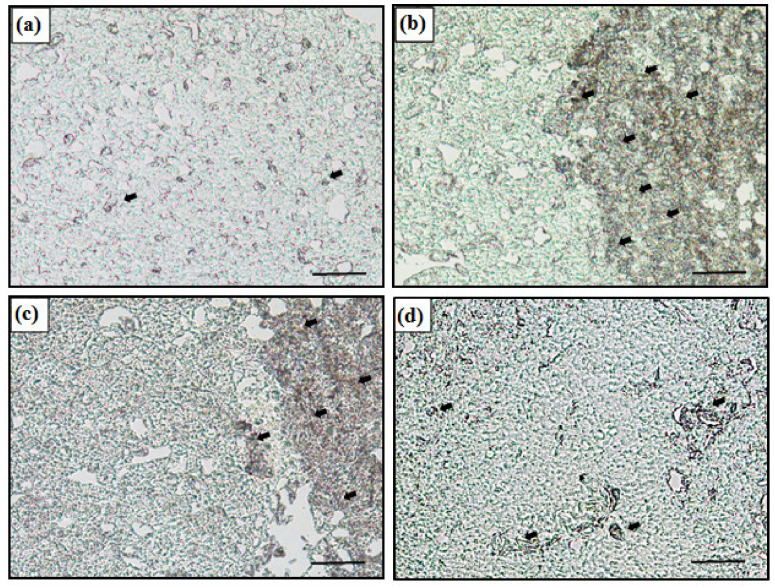
In situ hybridization of control and Zn 3.0 in 7 days. (**a**) Control, (**b**) Zn 3.0 + S, (**c**) Zn 3.0 + H, and (**d**) Zn 3.0 + V. The dark area (black arrow) indicates the mRNA expression of the metallothionein gene in the liver (Scale bars = 200 μm).

The mRNA expression of the metallothionein gene in liver was detected by in situ hybridization (Figure 2). In the figure, the dark area (black arrow) indicates the MT mRNA expression in the cytoplasm. The control showed no signal while the Zn-exposed groups expressed MT mRNA. Moreover, Zn 3.0 + S showed more signal than Zn 3.0 + H and Zn 3.0 +V, and the signal in Zn 3.0 + V was less than Zn 3.0 + H.

### 3.5. Changes in iNOS mRNA Expression in the Liver

**Figure 3:**
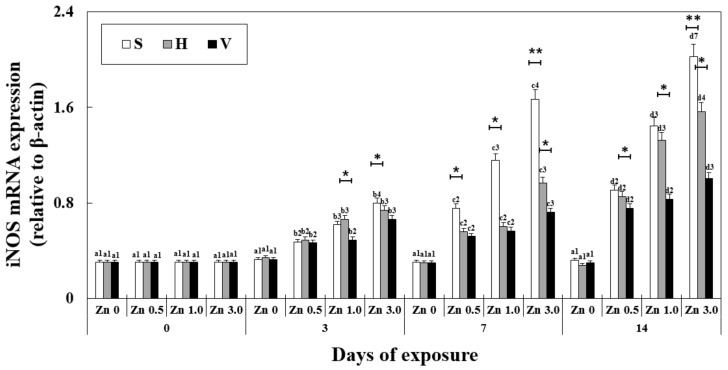
Changes in the mRNA expression of iNOS measured for 14 days. Different letters indicate significant differences among goldfish exposed to the same Zn concentration and water hardness for different exposure times (*p* < 0.05). Different numbers indicate significant differences among goldfish exposed to different Zn concentrations at the same water hardness level and for the same duration. The symbol “*” and “**” represent statistical significance (*p* < 0.05 and *p* < 0.01, respectively) among goldfish exposed to different water hardness levels at the same Zn concentration and for the same duration. Values are mean ± SD (*n* = 4).

iNOS mRNA expression was significantly affected by exposure time, Zn concentration, water hardness, and their interactions (Table 2). The changes in iNOS mRNA expression in the liver showed a similar tendency to MT (Figure 3). After 7 days in Zn 3.0 + S, the expression was 1.66 ± 0.2, which was 5 times that of the control (0.3 ± 0.04) at the same exposure time. At the same Zn concentration and exposure time, the increasing hardness of water decreased iNOS mRNA expression (2.02 ± 0.2 in Zn 3.0 + S, 578% of Zn 0 + S and 1.00 ± 0.1 in Zn 3.0 + V, 287% of Zn 0 + V on day 14). iNOS mRNA expression increased with the increasing concentration of Zn at the same water hardness environment and the same exposure duration.

### 3.6. Changes NO Activity and TAC in Plasma

**Figure 4:**
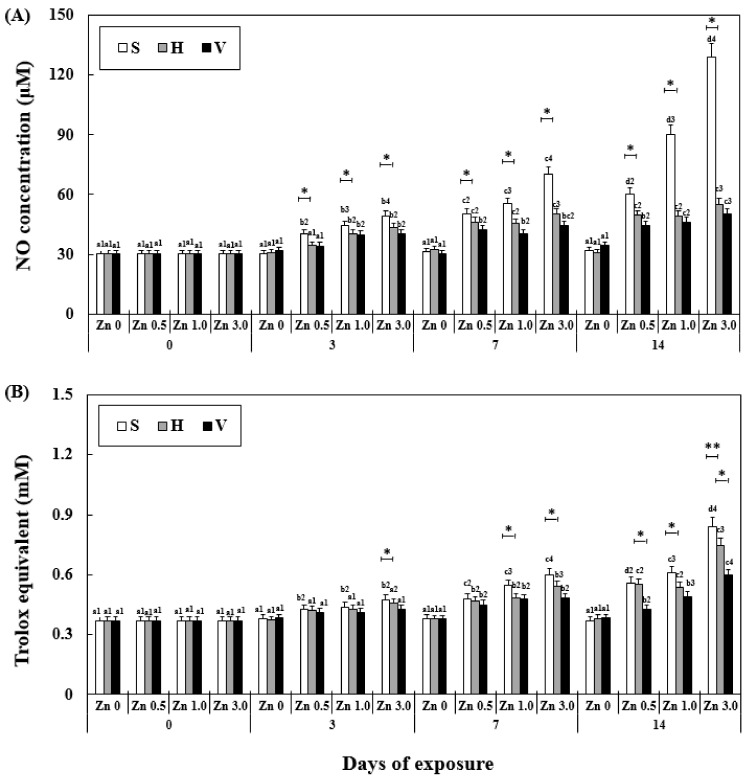
Changes in the NO (**A**) activity and TAC (**B**) measured for 14 days. Different letters indicate significant differences among goldfish exposed to the same Zn concentration and water hardness for different exposure times (*p* < 0.05). Different numbers indicate significant differences among goldfish exposed to different Zn concentrations at the same water hardness level and for the same duration. The symbol “*” and “**” represent statistical significance (*p* < 0.05 and *p* < 0.01, respectively) among goldfish exposed to different water hardness levels at the same Zn concentration and for the same duration. Values are mean ± SD (*n* = 4).

NO activity and TAC were significantly affected by exposure time, Zn concentration, water hardness, and their interactions (Table 2). At the same water hardness level, the NO activity increased with the increase in exposure time and Zn concentration. An increase in the NO activity with time occurred in the soft water group, and reached the highest among all the experimental groups (129.23 ± 9.8 μM). However, in the hard water and very hard water groups, the increase in NO activity was slower than that in the soft water groups, and the activity even became constant in Zn 3.0 + V between day 7 (44.21 ± 5.6 μM) and 14 (50.12 ± 4.9 μM). The TAC increased similar to the NO activity but not as rapidly, with less significant differences compared to the NO activity (Figure 4). Similar to the other stress factors in the experiment, TAC in the Zn 3.0 + S group exhibited the highest value (840.0 ± 70.0 μM, 426% of Zn 0 + S on day 14). 

### 3.7. Changes in Caspase-3 Activity in the Liver

**Figure 5:**
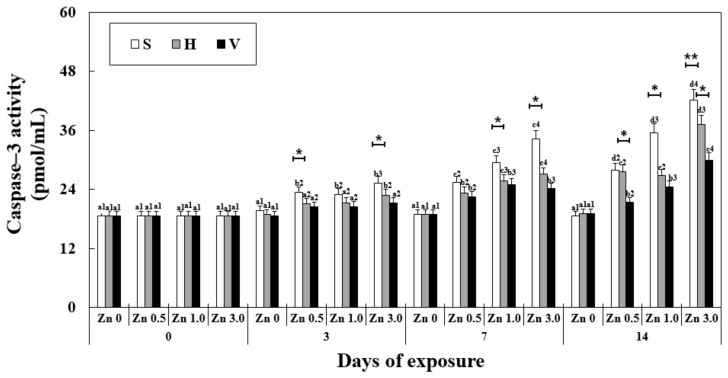
Changes in the activity of caspase-3 measured for 14 days. Different letters indicate significant differences among goldfish exposed to the same Zn concentration and water hardness for different exposure times (*p* < 0.05). Different numbers indicate significant differences among goldfish exposed to different Zn concentrations at the same water hardness level and for the same duration. The symbol “*” and “**” represent statistical significance (*p* < 0.05 and *p* < 0.01, respectively) among goldfish exposed to different water hardness levels at the same Zn concentration and for the same duration. Values are mean ± SD (*n* = 4).

The caspase-3 activity was significantly affected by the exposure duration, Zn concentration, water hardness, and their interactions (Table 2). A significant increase in caspase-3 activity in the liver was observed in the Zn-exposed groups from day 7 with the increase in the Zn concentration at the same water hardness level (Figure 5). On the 14th day, Zn 3.0 + S reached the highest expression among the groups in the experiment (42.12 ± 0.8 pmol/mL) and with the increase in the water hardness, the other groups showed a significant decrease in caspase-3 activity (37.22 ± 0.9 of Zn 3.0 + H, 88% of Zn 3.0 + S and 29.88 ± 1.0 of Zn 3.0 + V, 71 of Zn 3.0 + S). In all of the Zn 0 groups, no significant difference in caspase-3 activity occurred with the change in the water hardness levels.

### 3.8. TUNEL Assay

**Figure 6:**
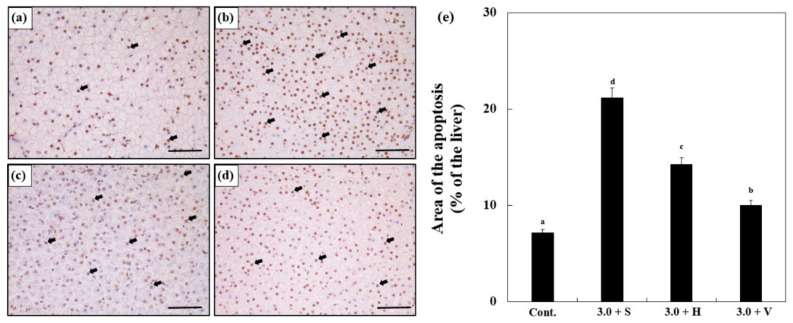
Images from a terminal deoxynucleotidyl transferase dUTP nick end labeling (TUNEL) assay using the liver cells of goldfish in the experiment for (**a**) control, (**b**) Zn 3.0 + S, (**c**) Zn 3.0 + H, and (**d**) Zn 3.0 + V. Brown cells indicate apoptotic cells. Scale bars = 200 μm. (**e**) Quantification of TUNEL assay images of the liver cells of goldfish exposed to different Zn concentrations and water hardness levels, which are indicated by the changes in the areas of apoptosis relative to the tissue area (%). Different letters indicate significant differences among goldfish exposed to different Zn concentrations and water hardness levels for the same duration. (*p* < 0.05). Values indicate means ± SD (*n* = 4).

The results of the TUNEL assay are shown in Figure 6 and the brown cells indicate the presence of apoptotic cells (Figure 6a–d). After 14 days, the Zn-exposed groups showed significantly more apoptotic cells than the control (7.11 ± 0.6%), and Zn 3.0 + S (21.14 ± 1.6%) showed more apoptosis than any other group. According to the results for Zn 3.0 + H (14.22 ± 1.0%) and Zn 3.0 + V (9.9 ± 0.7%), the numbers of apoptotic cells decreased with the increase in the water hardness (Figure 6e).

## 4. Discussion

Although Zn is an essential trace element in the fish body, high concentrations of Zn cause histological abnormalities, such as epithelial cell proliferation, necrosis, and mucus secretion [29]. However, previous research has reported that the lethal concentration (LC_50_) for the exposure of fish to heavy metals (including Zn) increased as the water hardness level increased [24]. Based on this finding, our research hypothesized that an increase in the water hardness could reduce the Zn toxicity stress generated in the body, and conducted a study to reveal this using the antioxidant measurement method. 

Therefore, in this study, after exposing goldfish to various concentrations of Zn and various water hardness levels for 14 days, we aimed to determine whether water hardness affected the reduction in Zn toxicity by comparing the experimental groups. 

During the entire experimental period, no significant changes were observed in the water hardness levels set at the beginning of the experiment. The results indicated that Ca^2+^ ions in the water did not decrease, and that Zn^2+^ ions did not significantly affect the change in the hardness of the water. However, the degree of Zn accumulation in the body increased as the exposure time elapsed with the increasing concentration of Zn. In contrast, as the water hardness increased, the degree of Zn accumulation in the body tended to decrease. 

Alsop and Wood [25] reported that the concentration of Zn^2+^ dissolved in the water decreased as the hardness of water increased. Hogstrand et al. [30] reported that Ca^2+^ and Zn^2+^ absorbed into the body decreased in the experimental group injected with CaCl_2_ as compared to the control injected with NaCl in rainbow trout, *Oncorhynchus mykiss*. 

As these findings are similar to the results of this study, we inferred that in higher water hardness groups, the Ca^2+^ ions present in large amounts in water not only reduced the solubility of Zn but also reduced its degree of absorption as Zn^2+^ is absorbed via the same pathway as Ca^2+^ ions through the gills.

In addition, the expression levels of MT and iNOS mRNA could determine the degree of change in toxic stress in the goldfish body based on the hardness and Zn exposure levels. On comparing hardness and similar Zn exposure time periods, the expression of the metallothionein gene and iNOS mRNA tended to increase in the high-concentration Zn experimental groups. Moreover, in the same experimental groups with the same Zn concentration, the expression levels were observed to increase as the hardness of water decreased. Furthermore, at the same water hardness level, the expression of MT and iNOS mRNA increased as the Zn concentration increased. According to a study by Khodadoust and Ahmad [14], when medaka, *Oryzias javanicus,* was exposed to Cd, a higher concentration of Cd showed higher expression levels of MT mRNA. In addition, Khan et al. [31] reported that the expression of NOS increased as a result of exposure to various heavy metals present in batteries in a fish species, *Oreochromis niloticus*. 

Consistent with the findings of previous studies, our study showed that the expression of the metallothionein gene and iNOS mRNA increased according to exposure to external heavy metals and toxic substances. Although Zn induced toxicity in the fish body, the toxic stress in the body was assumed to be reduced by Ca^2+^ ions by inhibiting the absorption of Zn into the body. 

In addition, the in situ hybridization results visually confirmed the mRNA expression of the metallothionein gene within the liver tissue of goldfish and showed that MT was expressed in the liver cytoplasm, with the lowest MT mRNA expression observed in the experimental groups treated with very hard water at the same Zn concentration. Macirella et al. [32] reported that after exposing *Denio rerio* to two different concentrations of Hg, MT expression was confirmed by in situ hybridization. They [32] also found that that MT mRNA expression was higher at a higher concentration of Hg exposure than that in the control, which occurred to protect against oxidation, and denatured the cells of proteins due to the toxicity of heavy metals. This was consistent with the results of our study. Goldfish, when exposed to Zn, activated a cell-protecting mechanism by increasing MT to alleviate the toxicity caused by heavy metals. The expression of the metallothionein gene in the liver cytoplasm of goldfish may have reduced because the very hard water environment reduced the effect of toxicity on heavy metals. 

In this study, the NO and TAC activities, which are representative oxidative stress indicators, were also measured to confirm the level of toxic substances that are generated in the body after exposure to various concentrations of Zn and hardness. We observed that the NO and TAC activity values did not significantly change with the change in hardness in the Zn 0 experimental groups. However, as the exposure time increased, the NO and TAC activities increased, and we observed that the NO and TAC activities decreased as the hardness increased among the high Zn concentration groups, whereas the NO and TAC activities increased as the Zn concentration increased. A previous study reported that the activity of various antioxidant proteins in the body were inhibited when goldfish were exposed to Zn concentrations of 0.1 and 1.0 mg/L [9]. Romano et al. [33] reported that no significant change was observed in the growth rate and metabolic activity of largemouth bass, *Micropterus salmoides*, at concentrations of 50 L to 600 mg/L, that is, for soft water to very hard water. 

Comparing the results of this study with those of the previous study [33], in the general freshwater environment without an additional supply of heavy metals, a change in the hardness value within 450 mg/L does not evidently have a significant effect on the stress response of the fish. In addition, under the water quality environment in which a 0.1 mg/L or higher concentration of Zn is present, Zn inhibited the antioxidant activity of *C. auratus* in vivo to increase the activity of RNS and ROS but suppressed Ca^2+^ ions in breeding water from being absorbed into the net. This consequently reduced the stress index of NO and TAC. To confirm the actual degree of apoptosis in the experimental section, caspase-3 activity was observed and a TUNEL assay was performed. Similar to the pattern of change in the previous stress indicators, the higher the Zn concentration, the higher the degree of caspase-3 activity and cell death. Zhao et al. [34] reported that exposing zebrafish *D. rerio* to 0–120 mg/L ZnO significantly increased the apoptosis in fish as the concentration of ZnO increased. Mo et al. [35] also reported that when HepG2 cells were exposed to Zn^2+^, caspase-3 activity and apoptosis were increased via the increase in ROS in the cells. 

Based on the previous studies, Zn may have induced the generation of free radicals, such as RNS and ROS, in the fish in this study, thereby increasing the degree of apoptosis. However, under the experimental conditions (hard and very hard water) with a high concentration of Ca^2+^ ions dissolved in water, the degree of caspase-3 activity and apoptosis decreased, and the higher the hardness, the higher the observed effect of apoptosis. Therefore, we conclude that a higher hardness level of water is effective in reducing the toxicological effect on Zn.

## 5. Conclusions

When goldfish were exposed to more than 0.1 mg/L of Zn, a toxic stress response was induced, but it was confirmed that this toxic stress was significantly reduced as the water hardness increased. Thus, we considered that Ca^2+^ ions in the water play an effective role in reducing heavy metals absorbed into the fish body. 

Moreover, to reduce the toxic stress that fish may be subjected to when exposed to heavy metal-contaminated environments, the appropriate hardness environment for various fish species must be studied further. In addition, investigating whether other water quality environmental factors, such as water temperature and pH, can reduce heavy metal toxicity through the ion absorption pathway of fish is valuable in this context. 

## Figures and Tables

**Table 1:** Primers used for qPCR amplification.

Genes (Accession No.)	Forward Primer	Reverse Primer
Metallothionein (X97271.1)	5′-TTA ACT GTG CCA CCT GC-3′	5′-AGG AAT TGC CCT TAC ACA CG-3′
iNOS (AY904362.1)	5′-AAG TCG TTT GCA TGG AGG AC-3′	5′-GGT GTC TAA GGT TGT TCA GG-3′
β-actin (LC382464)	5′-TTC CCT TGC TCC TTC CAC CA-3′	5′-TGG AGC CAC CAA TCC AGA CA-3′

**Table 2:** Interaction effects of Zn concentration and water hardness.

Time		Accumulation	MT	iNOS	NO	TAC	Caspase-3
Day 0	F	0.002	0.000	0.000	0.000	0.000	0.000
	*p*	0.998	1.000	1.000	1.000	1.000	1.000
Day 3	F	4.256	2.658	9.661	3.225	1.985	2.065
	*p*	0.045 *	0.084	0.010 *	0.052	0.152	0.149
Day 7	F	5.226	15.68	16.52	2.226	2.336	4.658
	*p*	0.032 *	0.002*	0.002 *	0.068	0.114	0.041 *
Day 14	F	29.28	28.10	5.662	9.658	3.521	7.632
	*p*	<0.001 *	<0.001 *	0.030 *	0.010 *	0.050 *	0.024 *

The symbol “*” indicates a significant difference.

**Table 3:** Zinc accumulation in the fish body (ppm).

Time	Hardness	Zn 0	Zn 0.5	Zn 1.0	Zn 3.0
Day 0	Soft	22.10 ± 1.54 ^a1^	21.91 ± 0.98 ^a1^	21.54 ± 1.66 ^a1^	22.10 ± 1.55 ^a1^
	Hard	22.00 ± 1.01 ^a1^	23.05 ± 1.39 ^a1^	21.80 ±1.26 ^a1^	21.75 ± 1.65 ^a1^
	Very hard	23.10 ± 1.18 ^a1^	22.65 ± 1.25 ^a1^	22.56 ± 0.99 ^a1^	22.25 ± 1.54 ^a1^
Day 3	Soft	22.18 ± 1.49 ^a1^	32.22 ± 2.22 ^b2^	37.18 ± 2.21 ^b2^**	40.22 ± 2.69 ^b2^**
	Hard	23.28 ± 1.95 ^a1^	29.09 ± 1.96 ^b2^	30.87 ± 1.96 ^b2^	34.45 ± 2.26 ^b3^*
	Very hard	23.79 ± 1.89 ^a1^	28.58 ± 2.01 ^b2^	28.91 ± 1.55 ^b2^	29.09 ± 1.14 ^b2^
Day 7	Soft	24.18 ± 1.22 ^a1^	41.27 ± 2.14 ^c2^**	48.57 ± 2.15 ^c3^*	57.39 ± 3.00 ^c4^**
	Hard	23.99 ± 1.36 ^a1^	36.15 ± 1.54 ^c2^*	48.48 ± 2.44 ^c3^*	49.60 ± 2.54 ^c3^*
	Very hard	23.14 ± 0.78 ^a1^	33.12 ± 1.33 ^c2α^	41.88 ± 2.01 ^c3^	40.33 ± 1.96 ^c3^
Day 14	Soft	23.47 ± 0.95 ^a1^	45.33 ± 1.92 ^d2^**	69.05 ± 3.24 ^d3^**	113.53 ± 5.94 ^d4^**
	Hard	30.12 ± 1.12 ^b1^*	40.97 ± 2.01 ^d2^*	54.54 ± 2.94 ^d3^*	90.07 ± 5.69 ^d4^*
	Very hard	24.48 ± 1.77 ^a1^	35.96 ± 1.87 ^c2^	47.59 ± 2.11 ^d3^	60.96 ± 4.36 ^d4^

Different letters indicate significant differences among goldfish exposed to the same Zn concentration and water hardness level. Different numbers indicate significant differences among goldfish exposed to different Zn concentrations at the same water hardness levels and for the same duration. The symbol “*” and “**” represent statistical significance (*p* < 0.05 and *p* < 0.01, respectively) among goldfish exposed to different water hardness levels at the same Zn concentration and for the same duration. Values are mean ± SD (*n* = 4).

## Data Availability

All relevant data are within the manuscript.
